# CHOPCHOP v3: expanding the CRISPR web toolbox beyond genome editing

**DOI:** 10.1093/nar/gkz365

**Published:** 2019-05-20

**Authors:** Kornel Labun, Tessa G Montague, Maximilian Krause, Yamila N Torres Cleuren, Håkon Tjeldnes, Eivind Valen

**Affiliations:** 1 Computational Biology Unit, Department of Informatics, University of Bergen, 5008 Bergen, Norway; 2 Mortimer B. Zuckerman Mind Brain Behavior Institute, Department of Neuroscience, Columbia University, New York, NY 10027, USA; 3 Sars International Centre for Marine Molecular Biology, University of Bergen, 5008 Bergen, Norway

## Abstract

The CRISPR–Cas system is a powerful genome editing tool that functions in a diverse array of organisms and cell types. The technology was initially developed to induce targeted mutations in DNA, but CRISPR–Cas has now been adapted to target nucleic acids for a range of purposes. CHOPCHOP is a web tool for identifying CRISPR–Cas single guide RNA (sgRNA) targets. In this major update of CHOPCHOP, we expand our toolbox beyond knockouts. We introduce functionality for targeting RNA with Cas13, which includes support for alternative transcript isoforms and RNA accessibility predictions. We incorporate new DNA targeting modes, including CRISPR activation/repression, targeted enrichment of loci for long-read sequencing, and prediction of Cas9 repair outcomes. Finally, we expand our results page visualization to reveal alternative isoforms and downstream ATG sites, which will aid users in avoiding the expression of truncated proteins. The CHOPCHOP web tool now supports over 200 genomes and we have released a command-line script for running larger jobs and handling unsupported genomes. CHOPCHOP v3 can be found at https://chopchop.cbu.uib.no

## INTRODUCTION

The use of CRISPR–Cas is now ubiquitous in modern molecular biology. First introduced as a tool for introducing repair-induced mutations in the genome, the emergence of catalytically dead or fused versions of the effector proteins has transformed CRISPR–Cas into a general purpose tool for targeting. For instance, CRISPR–Cas has been used to introduce new sequences into the genome (1–3), activate (4,5) or repress (6) transcription, as an enrichment tool for sequencing (7,8), for targeted hypermutation (9), as a diagnostic tool (10), to perform whole-organism lineage tracing (11), to target RNA molecules for destruction (12) or editing (13), and to track transcripts in live cells (14).

All CRISPR–Cas applications use a sgRNA to direct the CRISPR effector (Cas) protein to its target. In theory, CRISPR–Cas targeting only requires complementarity between the sgRNA and its nucleic acid target, but a number of studies have shown that efficient targeting follows more complex rules (15–21). For instance, the position of specific nucleotides in the target sequence, the accessibility of the target site, and the sequence of its flanking regions can all influence efficiency. The targeting efficiencies of Cas9 and Cas12a/Cpf1 have been measured in large-scale studies and combined with machine learning-based methods to optimize cutting (15,21).

There are other factors that can influence or prevent the generation of a null mutant. For instance, introducing a frameshift mutation too close to the start codon can permit translation initiation at a downstream ATG, leading to unintentional protein production. In other cases, targeting exons that are only present in a subset of isoforms can prevent null mutation generation. Finally, CRISPR–Cas gene editing can produce confounding phenotypes due to transcription adaptation or genetic compensation. For instance, a recent study showed that degradation of mutant mRNAs could result in the upregulation of related genes (22). In these situations, deleting the promoter can be a more robust method to produce knockouts.

Many CRISPR–Cas applications require the generation of a frameshift mutation to disrupt gene function, which requires a DNA repair event in which the number of inserted or deleted nucleotides is not a multiple of three. Surprisingly, it has been shown that double-strand break (DSB) repairs are not random: Cas9-induced DSBs using the same sgRNA often give rise to the same mutations (23). Recently, this has been incorporated into models that predict whether DSB repairs will give rise to a frameshifting mutation (24).

In summary, the CRISPR–Cas system has been adapted for a wide selection of uses, and numerous factors influence each of these modes, necessitating the existence of intuitive software for target selection. This new update of the CHOPCHOP web tool incorporates new CRISPR–Cas targeting modes and predicts frameshift mutation frequency in an improved, user-friendly interface.

## IMPROVEMENTS IN THE NEW RELEASE

**Figure 1.**
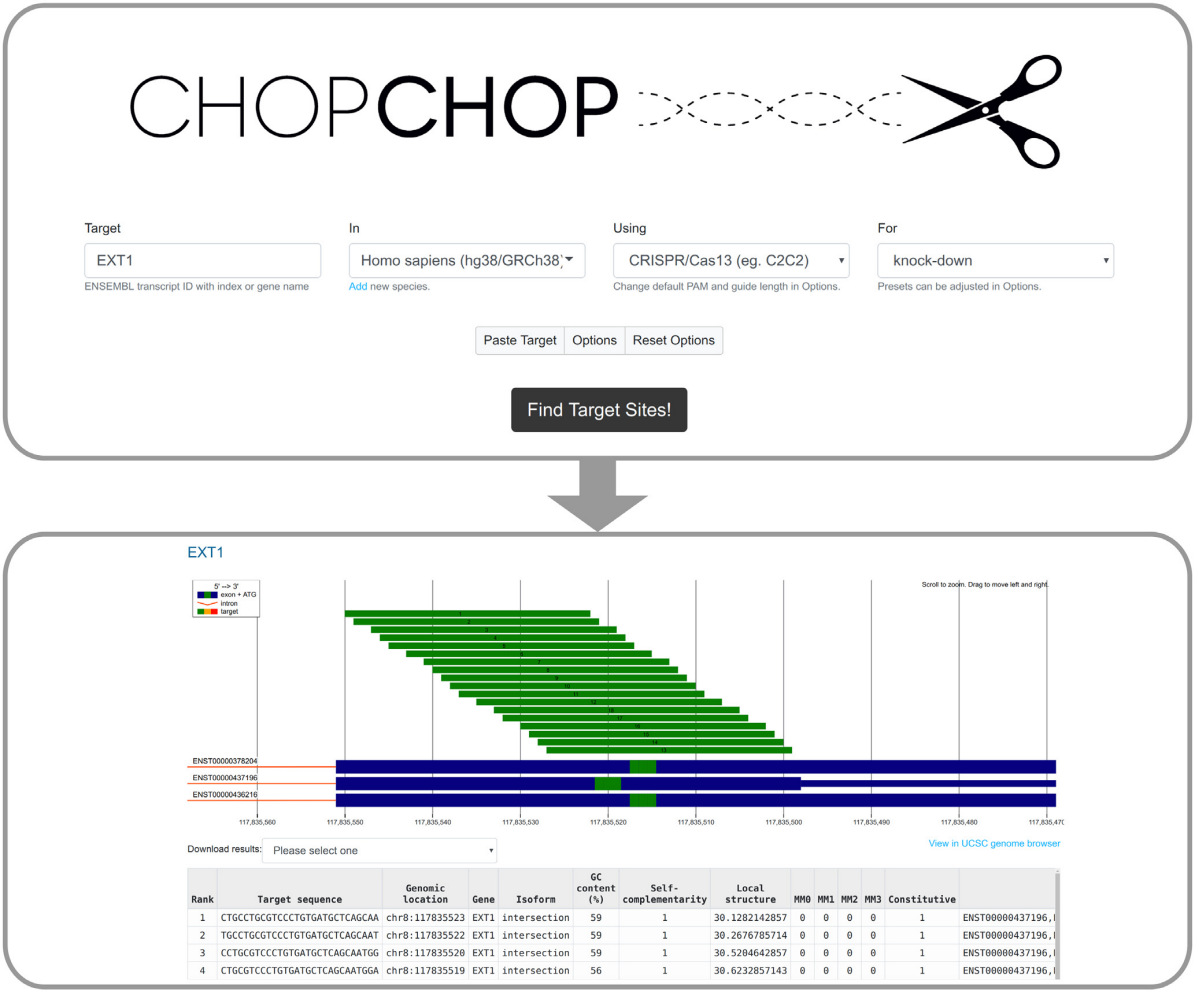
The workflow of CHOPCHOP when targeting RNA for knock-down. The CHOPCHOP homepage (upper box) require four types of input: (i) target, (ii) species, (iii) CRISPR effector and (iv) the purpose of the experiment. Default options will be adequate for most users, but advanced options can be revealed using the ‘Options’ button. The results of the search (lower box) are displayed along with all isoforms of the target gene. The target color indicates the quality of each sgRNA or nickase pair (green [best] to red [worst]). Below the graphic representation an interactive table allows for exploring each guide in greater detail.

Like previous versions, CHOPCHOP handles input from (i) gene and transcript identifiers, (ii) genomic coordinates and (iii) pasted sequences, and provides results in a number of output formats. The interface is simple (Figure 1) and requires the user to make four selections in the default mode: (a) target gene/isoform/region, (b) organism, (c) CRISPR effector (e.g. Cas9, CasX or Cas13), and (d) purpose (e.g. knockout, knockdown, repression). Advanced users can adjust the default settings by clicking the ‘Options’ button (Figure 1).

While CHOPCHOP queries typically run within a few seconds, heavy traffic can cause congestion during peak hours. We have therefore introduced a queuing system that ensures all users are prioritized. The new system retains results and calculations for up to 48 h after the initial query, permitting quick access (via caching) if an identical search is made later on and sharing results with collaborators. To increase speed for larger queries, CHOPCHOP also supports pre-filtering of sgRNA targets by (i) GC content (with a default of 10–90%) and (ii) the existence of self-complementarity within the sgRNA. This can greatly reduce computation time.

Several adjustments have been made to improve the results page visualizations. Notably, in-frame downstream ATGs are now colored in the isoform visualization (Figure 1) to help users avoid downstream translation initiation. In addition, the results table can now be sorted using any criteria.

In addition to these improvements, the latest CHOPCHOP introduces the following new major features.

### Targeting the transcriptome

The most widespread use of CRISPR–Cas is to introduce mutations into DNA. However, CRISPR–Cas systems have now been engineered to target RNA. For instance, CRISPR–Cas13 has been harnessed for transcript knockdown (25), live-cell transcript imaging (14) and RNA base editing (13).

CHOPCHOP now permits CRISPR–Cas13 targeting, and implements this functionality by searching for off-targets across the complete transcriptome rather than the genome. An important aspect of RNA targeting is to avoid regions of high structure that can reduce the accessibility of Cas13 (12,14,26). CHOPCHOP calculates RNA accessibility using RNAfold from the ViennaRNA package (27) according to published recommendations (14). Briefly, accessibility is calculated in windows of 70 nucleotides, obtaining the probability that a given nucleotide position in the transcript is unpaired. For each target, we take the mean probability of structure across each position targeted by the sgRNA (14).

Similar to protospacer adjacent motifs (PAMs) in DNA-targeting modes, we support any 5′ or 3′ protospacer flanking sequences (PFS) for RNA targeting. The Cas13a PFS - an H at the 3′ end of the target - is the default. As with the PAM, the PFS should be present in the sequence of the DNA or RNA target, but not in the sgRNA. CHOPCHOP provides the appropriate sgRNA sequence, ensuring that the user does not include the PFS when ordering the sgRNA oligonucleotide.

### New modes for targeting the genome

CHOPCHOP v3 expands the number of modes for DNA targeting. These include: (i) Nanopore enrichment mode. Targeted sequencing is a method used to attain high quality sequencing reads in a specific region of interest. PCR-based methods for enriching genomic regions have some limitations, for instance the maximum length of the region that can be enriched. By contrast, CRISPR–Cas provides a powerful method to excise genomic regions prior to amplification and sequencing with Oxford Nanopore Technologies (ONT) (7,8). The nanopore enrichment mode in CHOPCHOP allows users to identify pairs of gRNAs flanking large regions (up to 40kb) by excluding low-efficiency guides. The mode filters all sgRNAs with predicted self-complementarity, as this can have inhibitory effects on global Cas9 activity (8,28 and ONT personal communication); (ii) Knock-in mode. This identifies the same sgRNAs as the knockout mode, but designs homology arms up to 2 kb, which, based on recent studies (1–3) can be used for targeted insertions; (iii) Activation/repression modes. These modes are designed for use with Cas9 fusion proteins with the intention of activating or repressing a gene. Specifically, the modes target the promoter region and its flanking sites according to the guidelines specified in (29–31) in order to bring the activating/repressing domain into close proximity with the transcription start site.

For the Cas9 knockout mode, we now create a prediction of DSB repair outcomes (24). The model estimates the probability that a given sgRNA will result in a frameshift mutation. In addition, we have added efficiency scores for Cas12a/Cpf1 (21) and updated the ‘Doench’ efficiency score to the newest version (15), which is now the default scoring metric for Cas9 genomic targeting.

### Expansion of genomes and targeting

CHOPCHOP now supports over 200 genomes and includes gene annotations for genomic targets, as well as three transcriptomes for RNA knockdown (human, mouse and zebrafish). While previous versions of CHOPCHOP required the selection of a specific isoform for targeting, this new version allows the user to target the entire gene. In its default ‘intersection’ mode, CHOPCHOP v3 only searches for sgRNAs present in every isoform (Figure 1). This mode can be disabled by selecting the ‘union’ mode, which will display all sgRNAs in all transcripts, as well as a column indicating whether the sgRNA targets a constitutive exon.

### Command-line version

In addition to the web interface, we also provide the code for the command-line version of CHOPCHOP, which can be run locally and is suited for larger queries or screens. This tool includes all of the functionality of the web interface in addition to extra functionality for larger experiments, such as the ability to design control sgRNAs that do not match any sequence in the genome. The command-line version of CHOPCHOP is compatible with ampliCan, a tool for sequencing-based assessment of mutations (32).

## DISCUSSION AND FUTURE DEVELOPMENTS

Since its inception, the CRISPR field has undergone constant and rapid innovation, requiring the parallel development of bioinformatic tools that accommodate the new findings and technologies. In just a few years, CRISPR–Cas has become a powerful targeting tool for silencing and activating both DNA and RNA in a range of contexts, each of which requires the application of specific rules. The latest release of CHOPCHOP addresses this challenge by adding new functionalities that reflect the ever-expanding CRISPR toolbox. So far, we have accommodated the favorite species of over 200 research groups, and as we continue to improve the functionality of CHOPCHOP, we will continue to accommodate new transcriptomes and genomes.

In conclusion, this major update expands the CHOPCHOP toolbox, retaining its position as one of the most easy-to-use, versatile CRISPR–Cas targeting tools available.

## DATA AVAILABILITY

The CHOPCHOP (version 3) server is available at https://chopchop.cbu.uib.no; the python code for local installation is available at https://bitbucket.org/valenlab/chopchop.
